# Favorable response to carbamazepine therapy in genetically proven myoclonus-dystonia child

**DOI:** 10.1186/s13052-021-00986-w

**Published:** 2021-02-15

**Authors:** Mohammed F. Aljabri, Naglaa M. Kamal, Abdulrhman Alghamdi, Hamdan Alghamdi, Naif Alomairi

**Affiliations:** 1 Alhada Armed Forces Hospital, Head of Pediatric Neurology Unit and Neurophysiology Department, Taif, Kingdom of Saudi Arabia; 2 Department of pediatrics and pediatric hepatology, Faculty of Medicine, Cairo University, Cairo, Egypt; 3 Department of pediatrics, Alhada Armed Forces Hospital, Taif, Kingdom of Saudi Arabia; 4 Department of internal medicine, Taif University, Taif, Kingdom of Saudi Arabia

**Keywords:** Myoclonus-dystonia, Sarcoglycan gene, Carbamazepine

## Abstract

**Background:**

Myoclonus dystonia (MDS) is a dominantly inherited genetic disorder caused by loss-of-function mutations in the epsilon sarcoglycan gene (SGCE).

**Case presentation:**

We here in report a twenty months old Saudi boy who presented to us with a concern that the child is unable to walk properly. On assessment, he was flexing his left arm and left leg that usually followed by a back-ward fall. Diagnosis of dystonia induced with initiation of movement was suggested that later on proven genetically to be pathogenic mutation of sarcoglycan gene. Carbamazepine therapy was initiated with dramatic response. Response was maintained at 4 years follow up.

**Conclusions:**

Our patient and the other previously reported cases might highlight the response of SGCE mutations to carbamazepine therapy.

**Supplementary Information:**

The online version contains supplementary material available at 10.1186/s13052-021-00986-w.

## Introduction

Myoclonus dystonia (MDS) is extremely rare and under diagnosed problem; often missed as movement disorder [1]. It is characterized by mild to moderate dystonia along with ‘lightning-like’ myoclonic jerks [2]. It results from loss-of-function mutations in the epsilon-sarcoglycan gene (SGCE) gene coding for an integral membrane protein found in both neurons and muscle fibers [2].

Those suffering from this disorder exhibit symptoms of rapid, jerky movements of the upper limbs (myoclonus), as well as distortion of the body’s orientation due to simultaneous activation of agonist and antagonist muscles (dystonia) [3].

MDS is inherited in an autosomal dominant pattern, however SGCE is an imprinted gene [3], so only the paternal allele is expressed. Therefore, children suffering from this disease inherit the mutation from their fathers. If the mutated allele is inherited from the mother, the child is not likely to exhibit symptoms [4].

This disorder is characterized by two primary features; myoclonus and dystonia with substantial variation in severity. The myoclonus component is often the primary and most disabling feature [2].

The presentation of MDS includes; rapid contractions of myoclonus alongside the abnormal postures of dystonia, as well as neurological and psychiatric issues [5]. This disorder typically begins during childhood with symptoms of myoclonus and slight dystonia, most commonly cervical dystonia or writer’s cramp [5].

Dystonia symptoms tend to not get exaggerated over the course of the disease and is rarely the only associated symptom, while the myoclonus symptoms can become more severe [5]. Psychiatric issues are clinically diagnosed with the afore mentioned symptoms and include depression, anxiety, personality disorders and addiction. Obsessive-compulsive disorder is associated with MDS as both have been found to have a commonality on chromosome 7 in various studies [5].

Neurological symptoms are relatively common in those with MDS. Multiple parts of the brain have been pinpointed to be affected by the disease including the brainstem, neocortex, pallidum, and thalamus causing the various symptoms including posture changes and tremors, and very rarely dementia [6].

## Case presentation

A 20 months old Saudi Boy presented to the pediatric neurology clinic with two weeks history of inability to walk properly as per his mother’s description. His standing and walking were interrupted with backward twisting and flexion of the left arm and left leg with the whole body seems to be in a forward flexion, his right arm having short flexion that do not sustain these movements would ultimately flowed by a backward fall; Video 1. For the last two weeks he was not able to return to his normal standing and walking.

Back to his perinatal history; he was a product of full-term uneventful pregnancy to apparently heathy parents with significant obstetric history as his mother had previous three abortions with no living siblings. His father died 6 months prior to presentation in a road traffic accident which added more to the tragedy of the family.

Regarding his past medial history, it was noncontributory with no previous hospitalizations or surgeries.

Developmentally, he was up to his age. He started sitting, standing and waking at proper age and his abnormal posture and gait were just noticed two weeks prior presentation. His fine motor development was appropriate, and he was able to use his hands appropriately in transferring things between them and he could use the spoon. His speech was also matching to his age.

His physical examination at the time of initial evaluation at rest the child was is fully conscious with full orientation, no clear cranial nerve affection. His four limbs had normal tone power and reflexes.

Twenty-four hours video EEG recording revealed no convulsive movements and no epileptogenic activities. His MRI brain without contrast showed normal brain structure and myelination. Inner ear functions were also assessed by specialist with no abnormalities detected.

### Genetic testing

Whole exome sequencing (WES) was requested. More than 20,000 genes of the patient’s DNA were enriched and sequenced. Filtering of the exome data targeted recessive, X-linked, and dominantly inherited diseases.

**Fig. 1:**
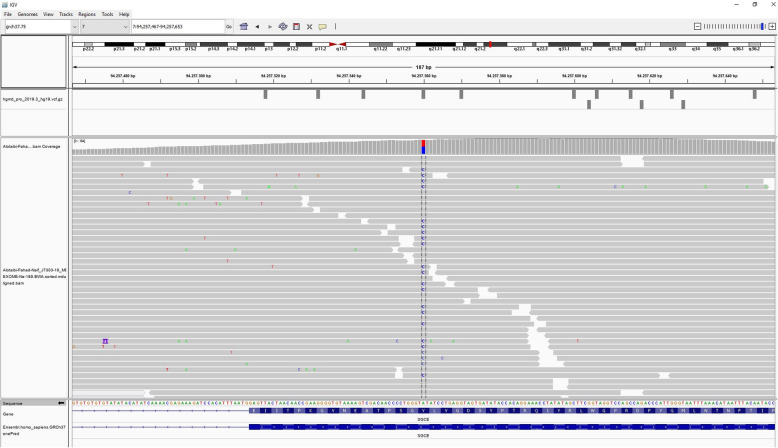
Chromatogram of the WES testing of the patient

WES identified the heterozygous variant c.344A > G, p.(Tyr115Cys) in SGCE which lead to an amino acid exchange (Fig. 1). Ten out of the 10 bioinformatic in silico program predict a pathogenic effect for this variant [7].

Allele frequency of this variant in the general population has not been documented. Considering the available information, the variant was classified as pathogenic. This variant causes autosomal dominant myoclonic-dystonia-11 (DYT11; OMIM#159900) [7].

The condition was explained to the mother and she was tested to the same mutation detected in her child which came out to be negative.

Management plan and follow up:

The abnormal movements are remarkably initiated with his intention to start walking so initial thinking was the possibility of paroxysmal kinesigenic dyskinesia (PKD).

Based on the chance that sodium pump inhibitors like phenytoin and carbamazepine might help in PKD, we started our patient on oral carbamazepine at an initial dose of 10 mg per kilogram per day which was doubled after a week time.

The patient was reviewed in the clinic after three weeks of implementing carbamazepine therapy with nearly complete resolution of the dystonia and was able to walk properly; Video 2.

Unfortunately, the mother was concerned by the side effect of the prolonged use of carbamazepine. She ignored medical advice and went to a local herbal and religious Sheik who advised her to stop the medication.

The patient had a complete relapse of his symptoms after withdrawal of carbamazepine. The child was readmitted, and carbamazepine was resumed.

On regular follow up over a period of 4 years, the child-maintained response on 20 mg/kg/day of carbamazepine therapy with minimal fine motor tremor in both hands without relapses.

The main concern of the mother was; for how long her child would continue on this medication? Would it be safe to wean him off carbamazepine?

The family on January 2020 decided to wean off the carbamazepine over one month and fortunately, there was no relapse so far.

## Discussion

The current recommendation for established diagnosis of MDS in pediatric age group is not exactly agreed on it. However, there are reports describing a favorable effect of, levetiracetam, clonazepam and valproate as single medication or in combination with trihexyphenidyl [1].

Trihexyphenidyl was reported to be effective alone in one 19 years old patient [8]. Trihyxaphendyl may not be safe and effective in infantile age group [9].

Deep brain stimulation is agreed to be considered as a modality for treating this disorder when proposed previous oral medication failed [10].

Carbamazepine therapy in combination to trihexyphenidyl was described in literature to be effective in alleviating the symptoms of MDS in one patient by Ghosh and his colleagues in 2013 [1].

Carbamazepine was the sole therapy to improve the symptoms of MDS for one patient after failure of several medications that include tetrabenazine, levetiracetam and clonazepam [11].

Fortunately, carbamazepine was the only medication implemented for our patient on the assumption that this dystonia is happening on the initiation of movement and the possibility of PKD.

Carbamazepine is a sodium channel blocker. It binds preferentially to voltage-gated sodium channels in their inactive conformation, which prevents repetitive and sustained firing of an action potential [12].

The primary symptoms of MDS include dystonia and myoclonus [1]. The myoclonus in our patient was documented not to be having cortical extension through video EEG which demonstrated that cortical recording was not interrupted at the time of movement added to that no clear epileptic discharges or slowing. The agreement is that this myoclonus is of non-cortical origin (peripheral myoclonus) [13].

Cortical myoclonus assumed to be worsened by sodium channel blocker that include carbamazepine and phenytoin [1].

Peripheral myoclonus is the terminology dedicated to any myoclonus that have an origin of subcortical. MDS of DYT11 may be considered part of it and consequently carbamazepine is not contraindicated in this type [1].

The dystonia is considered as segmental dystonia because it involves more than one part of the body [14]. In our patient it involved the cervical region and trunk.

Dystonia is expected to originate subcortical particularly in the basal ganglia [14]. The origin of organized motor activities was non pathological as the basal ganglia was not damaged as shown in patient’s MRI. Since the sarcoglycan gene was defective; it might lead to inappropriate transmission of basal ganglia orders resulting in dystonia. This is only hypothesis that need to be justified by further studies.

carbamezapine was shown to exerts different mechanism of action on serotonin [11] and on anti-diuretic-hormone that may have an impact on the dystonic symptoms [12].

## Conclusion

Carbamazepine might be a good choice to improve MDS symptoms. This assumption needs to be validated with multicenter double-blind-placebo-controlled trials but owing to the extreme rarity of this condition those studies may not be feasible.

## Supplementary Information

**Additional file 1.**

**Additional file 2.**

## Data Availability

All data and materials related to the study are included in the current manuscript.

## Abbreviations

MDS: Myoclonus dystonia
SGCE: Epsilon sarcoglycan gene
EEG: Electroencephalogram
MRI: Magnetic resonance imaging
WES: Whole exome sequencing
PKD: Paroxysmal kinesigenic dyskinesia
DYT11: Autosomal dominant myoclonic-dystonia-11
